# Insect pests of sweetpotato in Uganda: farmers’ perceptions of their importance and control practices

**DOI:** 10.1186/2193-1801-3-303

**Published:** 2014-06-24

**Authors:** Joshua Sikhu Okonya, Robert OM Mwanga, Katja Syndikus, Jürgen Kroschel

**Affiliations:** Global Program of Integrated Crop and Systems Research, International Potato Center (CIP), Plot 47, Ntinda II Road, Naguru, Box 22274, Kampala, Uganda; International Potato Center (CIP), Plot 47, Ntinda II Road, Naguru, Box 22274, Kampala, Uganda; IMOswiss AG, Weststrasse 51, CH-8570 Weinfelden, Switzerland; Global Program of Integrated Crop and Systems Research, International Potato Center (CIP), Avenida La Molina 1895, Lima, 12 Peru

**Keywords:** Sweetpotato butterfly, *Acraea acerata*, African sweetpotato weevils, *Cylas puncticollis*, *Cylas brunneus*, Production constraints, *Ipomoea batatas*, Integrated pest management

## Abstract

Insect pests are among the most important constraints limiting sweetpotato (*Ipomoea batatas*) production in Africa. However, there is inadequate information about farmers’ knowledge, perceptions and practices in the management of key insect pests. This has hindered development of effective pest management approaches for smallholder farmers. A standard questionnaire was used to interview individual sweetpotato farmers (n = 192) about their perception and management practices regarding insect pests in six major sweetpotato producing districts of Uganda. The majority (93%) of farmers perceived insect pests to be a very serious problem. With the exception of Masindi and Wakiso districts where the sweetpotato butterfly (*Acraea acerata*) was the number one constraint, sweetpotato weevils (*Cylas puncticollis* and *C. brunneus*) were ranked as the most important insect pests. Insecticide use in sweetpotato fields was very low being highest (28–38% of households) in districts where *A. acerata* infestation is the biggest problem. On average, 65% and 87% of the farmers took no action to control *A. acerata* and *Cylas* spp., respectively. Farmers were more conversant with the presence of and damage by *A. acerata* than of *Cylas* spp. as they thought that *Cylas* spp. root damage was brought about by a prolonged dry season. Different levels of field resistance (ability of a variety to tolerate damage) of sweetpotato landraces to *A. acerata* (eight landraces) and *Cylas* spp. (six landraces) were reported by farmers in all the six districts. This perceived level of resistance to insect damage by landraces needs to be investigated. To improve farmers’ capabilities for sweetpotato insect pest management, it is crucial to train them in the basic knowledge of insect pest biology and control.

## Introduction

Sweetpotato (*Ipomoea batatus* L. Lam.) is the world’s sixth most important food crop consumed after rice (*Oryza sativa L.*), wheat (*Triticum aestivum* L.), potato (*Solanum tuberosum* L.), maize (*Zea mays* L.), and cassava (*Manihot esculenta* Crantz) (CIP 2010). It is also the third most important root crop grown in eastern Africa after cassava and potato (FAO 2011). Sweetpotato is both a staple and a food security crop in eastern and southern Africa, and is mainly grown by smallholder women farmers (Mutuura et al. 1992; Bashaasha et al. 1995; Andrade et al. 2009). Sweetpotato is also grown for its vines as planting material; leaves are often eaten as a vegetable while shoots and roots are used as animal feed in many countries. In Uganda and western Kenya, the sale of fresh sweetpotato roots, vines and processed foods in both local and urban markets is becoming increasingly popular thus contributing to household cash income (Abidin 2004; Kaguongo et al. 2012). Orange-fleshed sweetpotato is also a rich source of beta-carotene, a precursor of bio-available vitamin A, and has potential of combating Vitamin A deficiency among rural resource-constrained farmers in many developing countries (Jalal et al. 1998; Jaarsveld et al. 2005; Low et al. 2007; Mwanga et al. 2003a; Burri 2011).

Sweetpotato production in Uganda declined from 2.84 million tons in 2010 to 2.55 million tons in 2011 (FAO 2011). Uganda also dropped from being the second to being the fourth largest producer of sweetpotato in the world after China, Tanzania and Nigeria (FAO 2011). This reduction in sweetpotato production could be due to many biotic stresses including insect pests and diseases (viral and fungal) that have led to the decline in vine and root quality and root yields. Research conducted in most southern and eastern Africa has shown that insects are among the most economically important pests of sweetpotato (Smit 1997). The most serious and commonly reported insect pest species in Uganda (Smit 1997; Hakiza et al. 2000; Abidin 2004; Ebregt et al. 2005), Kenya (Smit 1997; Nderitu et al. 2009), Ethiopia (Azerefegne et al. 2001), Rwanda (Smit et al. 1997) and Nigeria (Girma 1994; Tewe et al. 2003) are caterpillars of the sweetpotato butterfly, *Acraea acerata* Hew. (Lepidoptera: Nymphalidae), the African sweetpotato weevils, *Cylas brunneus* F. and *C. puncticollis* Boheman (Coleoptera: Brentidae), the clearwing moth *Synanthedon* spp. (Lepidoptera: Sesiidae), the sweetpotato hornworm, *Agrius convolvuli* L. (Lepidoptera: Sphingidae) and vectors of the sweetpotato virus diseases, such as the whitefly *Bemisia tabaci* Gennadius (Hemiptera: Aleyrodidae) (Fuglie 2007). *Acraea acerata* and *Cylas* spp. occur in 15 and 23 African countries, respectively (CAB International 2005).

The two African *Cylas* species often occur together in fields and cause huge yield losses of up to 100% (Girma 1994; Smit 1997; Chalfant et al. 1990). Root and stem damage by *Cylas* species is of great economic importance as it leads to a reduction in root yield, and root and vine quality. Due to egg laying and extensive larval tunneling by *Cylas* species, the plant produces bitter defense terpenes and phenolic compounds that make roots unsuitable for both human and animal consumption (Chalfant et al. 1990; Ames et al. 1996).

The *A. acerata* larvae feed on leaves of the sweetpotato plant and heavy infestations can lead to complete plant defoliation. Defoliation of young plants by *A. acerata* larvae can sometimes result in failure of the crop to re-establish and hence reduced yields. As a result, farmers continue to record huge losses due to these pests. Since sweetpotato is an important food security crop, its low production has a bearing impact on national food security. Understanding sweetpotato production constraints, particularly insect pests and farmers’ methods of managing *A. acerata* and *Cylas* species, could be useful in designing an effective integrated pest management (IPM) strategy. If sweetpotato IPM research is to be useful and sustainable, it is important to understand farmers’ perceptions and their management methods.

Assessing farmers’ perceptions of crop production constraints has been used as a tool for documenting pest status and designing pest management options suitable for a particular community (Smit and Matengo 1995; Obopile et al. 2008). Soleri et al. (2000) emphasized the need for integrating methods from both biological and social sciences to understand farmers’ selection criteria of crop varieties, since objectives of farmers while selecting a particular maize variety, differed from what research scientists had normally assumed. In a similar way, it would be equally important for crop entomologists to integrate farmers’ perspectives of insect pest management in the development of any intervention measure for local use. Bonhof et al. (2001) used farmer participatory rural appraisals for maize farmers at the Kenyan coast to understand the pest status and control strategies for the maize stemborer (*Chilo partellus* Swinhoe). In central Zambia, Mukanga et al. (2011) solicited farmers’ views on various management practices of maize ear rots caused by several fungal pathogens before resistant varieties to ear rots could be developed. Lebesa et al. (2012) and Midega et al. (2012) undertook household surveys in western Kenya using questionnaires to determine the pest status of herbivorous blister beetles (*Hycleus* spp.) and cotton (*Gossypium hirsutum* L.) pests. Tounou et al. (2013), similarly used questionnaires to interview farmers to determine geographic distribution and importance of stemborers on maize in southern Togo.

The amount of edible sweetpotato roots and vines damaged by insect pest infestations is significant and calls for an effective management strategy. To develop an integrated pest management strategy that is appropriate for resource poor farmers, information is needed on the current distribution, importance and control methods of sweetpotato pests in various agro-ecologies of Uganda. In Uganda, detailed studies on sweetpotato insect pests are limited. Since the farm surveys conducted by Bashaasha et al. (1995) between 1989 and 1992 in nine different agro-ecological zones, no recent quantitative surveys have been undertaken in the country. The pest status and distribution of key insect pests are expected to vary considerably with the current climate variability. There is, therefore, need to find feasible solutions to agricultural production constraints by incorporating farmer views into research for development programs.

The International Potato Center (CIP) through its global program of integrated crop and systems research seeks to develop an effective IPM strategy for key insect pests of sweetpotato according to climatic factors and pest severity. In this regard, a questionnaire was developed to capture the information and to document the present insect pest distribution, farmers’ knowledge and coping strategies to control insect pests of sweetpotato. This study was specifically carried out to 1) determine farmers’ perceptions of importance of sweetpotato insect pests and their distribution, 2) analyze the major sweetpotato production constraints and farmers’ coping strategies for the control of sweetpotato insect pests in various agro-ecologies of Uganda, 3) identify local sweetpotato landraces that have some field resistance to damage by *A. acerata* and *Cylas* spp.

## Materials and methods

### Study area

**Figure 1:**
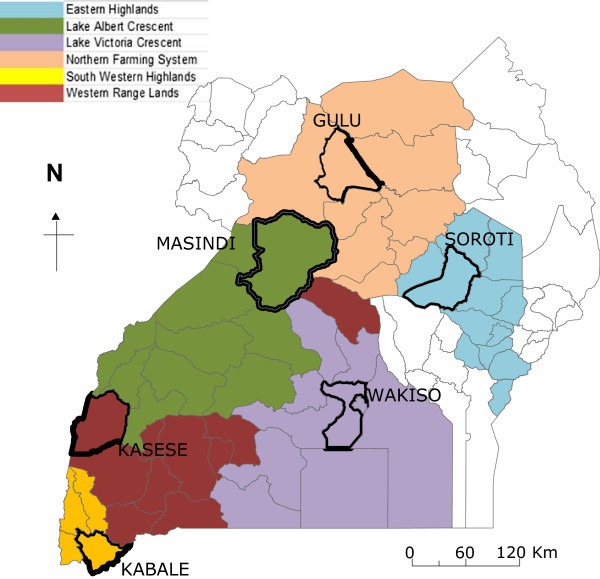
**Map of Uganda showing the six study districts with their corresponding agro-ecological zones; Gulu in the northern farming system, Masindi in the Lake Albert Crescent, Soroti in the Eastern Highlands, Wakiso in the Lake Victoria crescent, Kasese in the western range highlands and Kabale in the south western highlands.**

The study was conducted between August and October 2011 in six districts of Uganda. Major sweetpotato growing districts were purposively selected from six different agro-ecological zones to obtain a wide range of household perceptions. These were Gulu (2°46′48″N, 32°18′0″E), Kabale (1° 15′ 0″ S, 29° 59′ 24″ E), Kasese (0° 11′ 0″ N, 30° 5′ 0″ E), Masindi (1° 41′ 1″ N, 32° 43′ 20″ E), Wakiso (0°23′53″N, 32°28′41″E) and Soroti (1° 42′ 54″ N, 33° 36′ 40″ E) (Figure 1). In each district, two sub-counties were purposively selected based on intensity of sweetpotato farming as perceived by the district agriculture office for this observational study.

The questionnaire was pre-tested with five sweetpotato farmers in Luweero district, not included in the sample, three weeks before the study. After the pilot test, changes were made in the expression of some questions to be asked. The survey team consisted of two entomologists, one research assistant and twelve agricultural extension workers.

Farmers who had grown sweetpotato in 2011 and preferably still had it in their fields were randomly selected at regular intervals of 5–10 km distance between sites or until a sweetpotato growing household was found on motorable roads within the subcounties. A total of 32, both female and male farmers, were interviewed face-to-face in their homesteads in each district using a standard questionnaire – partly structured and partly open. All interviews were conducted in the local language of that district with the help of an enumerator. Data was collected on: sweetpotato cropping systems, constraints in sweetpotato production, sweetpotato insect pests and, their control measures and field resistance of sweetpotato varieties to the three major insect pests (*A. acerata, C. brunneus* and *C. puncticollis*).

Constraints to sweetpotato production were identified by asking farmers to mention all the constraints they had experienced, and ranking them in order of importance of each constraint (for instance most destructive, occurs frequently) in the last cropping season. Farmers were shown colored photographs and/or vials of insects (larval and adult stages) in alcohol to ensure they made the correct identification of the pest. Farmers’ compared the perceived field resistance (level of damage) of their presently cultivated local varieties to damage by the most important insect pests.

Quantitative data were subjected to analysis of variance (ANOVA) using General Linear Model (GLM) in SAS software (SAS Institute Inc 2008). Means were compared using Fisher’s LSD Test. Descriptive statistics (percentages and mean values) were the main statistical tools employed to analyze qualitative data.

## Results and discussion

### Farm household characteristics and some aspects of sweetpotato production

Sweetpotato production in the six districts of Uganda is a female dominated activity with males representing only 39% of 192 respondents. In Soroti district, however, male respondents out-numbered female respondents by 25%; this is probably because sweetpotato is more of a commercial than food security crop in this district. Men have been reported in many African countries to dominate cash crop production (World Bank et al. 2009). This domain change of sweetpotato, from being a female controlled crop to a male crop in Soroti due to commercialization, has been reported for other crops like coffee (*Coffea* spp.) in Uganda (Kasente et al. 2002) and cocoa (*Theobroma cacao* L.) in Ghana (Doss 2001). In Uganda, 51% of the population is female (UNHS 2010) and women are mostly responsible for household food crop production; in particular the sweetpotato crop which is referred to as a “female” crop (Bashaasha et al. 1995). Consequently, efforts geared towards promoting and disseminating new sweetpotato technologies should mainly target women.

Most of the sweetpotato farmers were in their active age (mean of 40 to 46 years) which is a good sign for food security in Uganda (Table 1). There is a huge potential to increase production if more investments are made into developing pest and disease resistant high yielding sweetpotato varieties as part of developing integrated pest management strategies. Mean household size across districts was 7.5 members; this is higher than the national average by 2.5 members (UNHS 2010). On average, Gulu district had the biggest households with 10 members, while Kabale district had the smallest household size of six members (Table 1).

**Table 1:** **Household characteristics of interviewed sweetpotato farmers in the six districts of Uganda and their sweetpotato production practices/techniques, August-October 2011**

Household characteristics	Gulu	Kabale	Kasese	Masindi	Soroti	Wakiso	p-value (N/A = not applicable)
Female respondents (%)	53.1	62.5	59.4	75.0	37.5	78.1	N/A
Female headed households (%)	28.1	28.1	21.9	28.1	15.6	43.8	N/A
No formal education (%)	28.1	25.0	18.8	9.4	6.3	20.0	N/A
≤7 years of formal education (%)	40.6	65.6	56.3	46.9	71.9	60.0	N/A
>7 years of formal education (%)	31.3	9.4	25.0	43.8	21.9	20.0	N/A
Mean elevation (m a.s.l.)	1091^cd^	2090^a^	1006^e^	1124^c^	1079^d^	1191^b^	<0.0001
Mean age of respondent (years)	43.7 ± 2.6^a^	40.0 ± 2.0^a^	41.2 ± 2.4^a^	44.9 ± 2.4^a^	44.1 ± 2.0^a^	45.8 ± 2.4^a^	0.7024
Mean household size (persons)	10.2 ± 1.3^a^	5.9 ± 0.5^c^	6.6 ± 0.5^bc^	6.5 ± 0.6^c^	8.6 ± 0.7^ab^	7.5 ± 0.8^bc^	0.0002
Mean rotation duration (months)	20.4 ± 2.2^ab^	11.0 ± 1.7^c^	6.6 ± 0.8^c^	17.2 ± 1.4^b^	23.3 ± 2.1^a^	9.1 ± 1.1^c^	<0.0001
Mean years of growing sweetpotato	25.1 ± 2.7^ab^	19.6 ± 1.9^abc^	16.8 ± 2.0^c^	26.3 ± 2.3^a^	23.8 ± 1.9^ab^	19.7 ± 2.4^bc^	0.0179
Mean sweetpotato acreage in 2011 (ha)	0.3 ± 0.1^ab^	0.2 ± 0.0^b^	0.2 ± 0.0^b^	0.2 ± 0.0^b^	0.3 ± 0.0^ab^	0.4 ± 0.1^a^	0.1393
Arable land devoted to sweetpotato (%)	12.3 ± 3.1^b^	30.5 ± 5.0^a^	17.9 ± 3.8^b^	14.1 ± 2.0^b^	20.0 ± 3.5^b^	31.1 ± 5.1^a^	<0.0001
Mean total land holding (ha)	100.2 ± 82.3^a^	2.9 ± 1.7^a^	2.4 ± 0.5^a^	6.6 ± 2.8^a^	2.4 ± 0.3^a^	1.3 ± 0.2^a^	0.2625
Total land cropped (%)	59.5 ± 6.3^c^	85.8 ± 5.6^ab^	85.4 ± 3.9^ab^	63.6 ± 5.9^c^	77.8 ± 5.6^b^	93.9 ± 3.0^a^	<0.0001

Means followed by the same letter in the same row are not significantly different (p ≥ 0.05, Fisher’s least significant difference). Values are means ± SE.

Mean elevation of the surveyed homesteads was highest in Kabale district (2090 m a.s.l.) and lowest in Kasese district (1005 m a.s.l.). Sweetpotato had been grown for more than 16 years in all the six districts. The average period of growing sweetpotato was longest (26 years) for Masindi followed by Gulu district (25 years). The average sweetpotato acreage in 2011 was highest in Soroti district (0.4 ha) and lowest in Kabale, Kasese and Masindi districts (0.2 ha). Sweetpotato is a commercial crop in Soroti district and this could explain the allocation of relatively larger portions of arable land to sweetpotato production.

### Constraints to sweetpotato production

Identification of factors limiting production and provision of environmentally-friendly options for integrated crop management is inevitable if sweetpotato production among smallholder farmers in Uganda is to be increased. In the districts visited, setbacks to sweetpotato production were many, however, insect pests attacking roots and leaves were the most important (Table 2). Poor yields of local varieties and degraded soils came second in importance while rodents or rats were ranked the third most important constraint. Other constraints that were unique to a district included delayed rainfall and weeds in Wakiso, shortage of land and small size of roots in Kabale, high cost of labor, floods, millipedes (Diplopoda) and poor roads in Kasese, extreme rainfall, high transport cost, lack of capital to plant large fields and high labor demands for the sweetpotato crop in Gulu districts.

**Table 2:** **Top five most important constraints to sweetpotato production as ranked by farmers in the study districts of Uganda, August-October 2011**

Constraints to sweetpotato production	Rank for each constraint (% households)*					
	1st	2nd	3rd	4th	5th	Mean
No constraint mentioned/experienced	0.5	8.3	26.6	55.7	81.8	34.6
Insect pests	33.9	42.2	33.3	18.8	5.7	26.8
Poor yields of local varieties/soils	12.0	10.4	6.3	1.6	1.6	6.4
Rats and rodents	8.3	8.9	6.3	4.2	1.0	5.7
Drought/prolonged dry seasons	10.9	3.1	4.2	2.6	0.5	4.3
High cost of labor/labor intensive/shortage of labor/weeds	6.3	2.1	2.6	1.6	1.6	2.8
Lack of market	3.6	5.2	2.1	2.1	0.5	2.7
Viral diseases	2.6	3.1	3.1	2.6	1.0	2.5
Shortage of planting material	5.2	1.6	3.1	1.0	0.0	2.2
Wild game (elephants, hippos, pigs)	4.2	2.6	1.6	1.0	1.0	2.1
Others	1.5	2.6	2.1	3.1	1.0	2.1
Millipedes	1.6	1.6	3.6	0.5	2.6	2.0
Land shortage	2.1	2.1	2.6	0.5	0.5	1.6
Floods/excess rainfall/storms	1.0	1.6	1.0	1.0	0.5	1.0
Lack of money to hire labor/plant large fields/build drying places	2.1	1.6	0.0	0.5	0.0	0.8
Domestic animals (cattle, goats)	1.0	1.0	0.0	1.0	0.5	0.7
Changed onset and cessation of rainfall in the seasons	1.6	0.5	0.5	0.5	0.0	0.6
*Alternaria* blight disease	1.0	0.5	0.0	1.0	0.0	0.5
Root rots	0.0	1.0	1.0	0.5	0.0	0.5

*Some columns do not add up to 100% due to rounding off.

### Sweetpotato insect pests

Among the sweetpotato insect pests reported by farmers, *Cylas* spp. were ranked number one by 87% of the households followed by caterpillars of *A. acerata* (60.7%) (Table 3). Caterpillars of *A. acerata* in Masindi and Wakiso districts were perceived to be more damaging to sweetpotato than the *Cylas* spp. Insect pests that appeared to be local included *Agrius convolvuli* in Gulu and Soroti districts, and the sweetpotato armyworm (*Spodoptera* spp.) in Soroti district. *A. convolvuli* occasionally appeared in both districts as in 2009 and 2010 where it caused serious sweetpotato damage leading to food insecurity (IPC 2010). *Spodoptera* spp. had occurred in Soroti in the past three consecutive years (2007 to 2010). Small black ants (“*Munyeera*” in Luganda) were mentioned to construct nests inside the stem base of sweetpotato plants leading to vine damage. Ants are not pests *per se* but rather predators of *Cylas* spp. larvae feeding inside the stem base.

**Table 3:** **Major insect pests experienced by farmers in sweetpotato (% households) and rank**

Insect pest reported	% households*						
	Gulu	Kabale	Kasese	Masindi	Soroti	Wakiso	Overall mean
Sweetpotato weevils (*Cylas* spp.)	97 (1)	56 (1)	84 (1)	100 (2)	94 (1)	91 (1)	87.0
Sweetpotato butterfly (*Acraea acerata*)	28 (2)	50 (2)	88 (2)	88 (1)	13 (2)	97 (2)	60.7
Sweetpotato hornworm (*Agrius convolvuli*)	33	0	0	0	25	0	9.7
Armyworm (*Spodoptera* spp.)	0	0	0	0	34	0	5.7
Others (ants, whiteflies)	0	0	9	3	3	0	2.5

*percentage values add to more than 100 due to multiple responses. Rank (in parentheses) for the top two most important insect pests.

Our results are in accordance with those reported by Girma and Belehu (1994) in Ethiopia where insect pests and specifically *A. acerata* and *Cylas* spp. are most important. Farmers reported that *A. acerata* was important only in some years with the last distinct outbreak in Soroti being in February 2010. Farmers in Masindi, however, said that *A. acerata* started to be a problem in 1993 until now.

It is important to note that farmers tend to have a high perception of damage caused by insect pests, hence, rank them highly (Smit 1997). This must have been the case in the current study since results from a subsequent field survey of farmers’ sweetpotato fields in Kabale, Buliisa and Masindi districts reported lower insect pest infestation rates and densities (Okonya and Kroschel 2013a, b). *B. tabaci* is an important insect vector of the sweetpotato virus disease component (*Sweetpotato chlorotic stunt virus*) in Uganda. However, most farmers did not consider *B. tabaci* an important insect pest because they do not have the knowledge to relate the presence of the insect (vector) to virus symptoms or crop damage and yield loss. Such knowledge gaps call for better training on IPM through the national extension services.

There was a general belief by farmers that once sweetpotato is attacked by *A. acerata*, root yield will be poor. The African locust (*Locusta migratoria migratorioides* (R. & F.) appeared in Soroti district in 2008/2009 cropping season and completely defoliated sweetpotato plants.

### Farmers management practices of sweetpotato pests

The main methods used by farmers in managing sweetpotato insect pests on their farms included chemical insecticide application, ash application, hand-picking or a combination of two or more physical and cultural strategies (Table 4). Control strategies for caterpillars of *A. acerata* included use of chemical insecticides of mainly permethrin, dimethoate and cypermethrin by 24% of the households; application of wood ash, hand-picking of caterpillars and a combination of two or three of the above mentioned methods. Early harvesting was the most common method used to limit the damage caused by *Cylas* spp., however, mulching, re-hilling to cover soil cracks, crop rotation and insecticide application were being used but on a very low scale. On average, 65% and 87% of the sweetpotato farmers did not control *A. acerata* and *Cylas* spp., respectively.

**Table 4:** **Control methods for the two (** ***A*** **.** ***acerata*** **and** ***Cylas*** **spp.) most important insect pests of sweetpotato (% households)**

Control strategy for *A*. *acerata*and *Cylas*spp.	% households						Overall mean
	Gulu	Kabale	Kasese	Masindi	Soroti	Wakiso	
**a) for** ***A. acerata***							
Chemical insecticides	2	20	14	50	0	56	24
Ash application	0	0	7	8	0	0	3
Hand-picking	0	0	0	8	0	6	2
Chemical insecticides and hand-picking	0	0	0	8	0	0	1
Chemical insecticides and ash application	0	0	0	0	0	13	2
Hand-picking and ash application	0	0	0	8	0	0	1
Chemical insecticides, hand-picking and ash application	0	0	0	0	0	6	1
None	98	80	79	17	100	19	65
**b) for** ***Cylas*** **spp.**							
Chemical insecticides	0	0	0	6	0	8	2
Chemical insecticides and re-hilling	7	0	7	6	0	0	3
Crop rotation	7	8	0	0	0	0	3
Early harvesting	0	0	7	0	7	8	4
Mulching and re-hilling	0	0	0	0	0	8	1
None	87	92	87	88	93	77	87

Use of chemical insecticides was relatively low; being highest in Wakiso district followed by Masindi district but absent in Soroti district (Table. 4). Insecticide application was highest in districts which ranked *A. acerata* as the main insect pest damaging sweetpotato. It was evident that some farmers implemented more than one control strategy to reduce field infestation by insect pests. Aiming at improving the effectiveness of available control methods is therefore desired. The low use of insecticides in sweetpotato could partly be due to the high cost of insecticides which subsistence farmers cannot afford but also lack of knowledge about pest biology of especially *Cylas* spp. Many farmers did not know how to control *Cylas* spp. However, farmers who applied insecticides to control *A. acerata* observed reduced damage by *Cylas* spp. as well. This therefore encouraged farmers to apply insecticides two months after planting even in the absence of *A. acerata*.

Due to the severity of pest infestation during outbreaks, farmers usually received insecticides from agriculture extension workers or local authorities to spray against *A. acerata* in Kabale district and *A. convolvuli* in Gulu and Soroti districts. Use of chemical insecticides against *A. acerata* could be because farmers took the pest seriously, pest and pest damage were more visible or pesticides are effective. It should be noted, however, that use of chemical insecticides is not a permanent solution as it can be disastrous to human health due to poor handling, elimination of natural enemies for the pest and is out of reach for most of the resource constrained poor farmers (Croft and Brown 1975).

### Major sweetpotato varieties grown and farmers perceptions of their field resistance to insect pests

Sweetpotato variety mixtures were a common practice by at least 89% of interviewed farm households. With the exception of households in Kabale and Soroti districts, farmers in the remaining districts sometimes planted vines affected by insect pests due to lack of planting materials, especially at the end of a prolonged dry season. The most popular grown varieties were Larila in Gulu, Rwabafuluki in Kabale, Rwatoro in Kasese, Dimbuka in Masindi, Kampala and Araka in Soroti and NASPOT 1 in Wakiso district (Table 5). Farmers identified eight landraces (Mukono, Kigabali/Magabali, Red mamba, Kiryenamwami, Dimbuka, Boy, Setyabule and Mbale), which have shown some form of field resistance to vine damage by *A. acerata* and six landraces (Ochol/Ocuc, Rwatoro, Muhamoud, Dimbuka, Kyebandula and Opaku) with some form of resistance to field root damage by *Cylas* spp. Information on resistance levels of landraces needs to be taken with caution since no variety has been reported to be resistant in laboratory no choice experiments to *Cylas* spp. for instance (Mwanga et al. 2003a, b). This notwithstanding, these reports of resistance of landraces to insects need to be investigated further as they may provide potential sources of resistance to these two most economically important pests of sweetpotato. Various authors have found differences in *Cylas* spp. damage among cultivars (Mwanga et al. 2003b; Stathers et al. 2003). Factors such as quantity of root latex, depth of rooting and amount of foliage have been reported to contribute to reduced *Cylas* spp. field damage (Mwanga et al. 2001; Stathers et al. 2003). Anyanga et al. (2013) found that chemical compounds in the root latex were responsible for the host plant resistance to *Cylas* spp. damage of “New Kawogo” sweetpotato variety. This variety (New Kawogo) was also mentioned by farmers in Masindi and Wakiso districts in this study to be resistant to *Cylas* spp. damage.

**Table 5:** **Perceived field resistance of major local sweetpotato varieties or landraces to** ***A*** **.** ***acerata*** **and** ***Cylas*** **spp. in 2011**

District	Local name of Sweetpotato variety/landrace	% Households growing the variety	Resistence level to *Acraea acerata*damage (% responses)			Resistence level to *Cylas*spp. damage (% responses)		
			Low	Moderate	High	Low	Moderate	High
GULU	Lalira	22	0	15	9	0	13	11
	Alero	12	0	6	3	0	7	4
	Ochol/Ocuc	12	0	6	3	1	7	1
	Adoch	11	0	6	15	0	2	9
	Mukiga	3	0	3	0	0	2	2
	Others	40	0	18	18	0	18	21
KABALE	Rwabafuluki, Kandazi/Mulungi	22	0	4	25	0	7	17
	Mukazi	17	0	0	11	0	17	14
	Mukono	8	4	0	11	0	7	3
	Kidodo	6	0	4	11	0	3	0
	Kigabali/Magabali	4	4	0	4	0	7	0
	Others	43	0	0	25	0	17	7
KASESE	Rwatoro	13	0	2	9	2	2	6
	Red mamba	10	2	4	4	0	4	0
	Rosemary	10	0	4	7	0	6	6
	Kiryenamwami	8	2	2	7	0	4	4
	Muhamoud	6	0	4	7	2	2	6
	Bitambi	6	0	2	2	0	2	2
	Kyebandula	6	0	2	2	0	4	2
	Others	41	11	0	26	4	21	17
MASINDI	Dimbuka	22	1	7	18	1	16	10
	Nakato/Nyakato	14	0	6	12	0	14	5
	Kahogo/New Kawogo	9	0	4	9	0	5	4
	Kyebandula	5	0	1	4	1	1	0
	Suwedi	3	0	3	1	0	3	3
	Kabakumba	3	0	0	3	0	0	3
	Others	44	0	4	25	0	18	16
SOROTI	Kampala	18	0	0	11	0	6	12
	Araka	18	0	5	16	0	8	12
	Ateseke	7	0	5	11	0	6	4
	Opaku	5	0	0	0	1	4	1
	Letesi/Latesi	4	0	0	0	0	3	1
	Mwambi	4	0	5	11	0	3	1
	Boy	4	5	0	0	0	0	4
	Others	40	0	16	16	1	16	17
WAKISO	Naspot 1	31	0	16	23	0	18	35
	Dimbuka	23	2	5	16	3	3	15
	Setyabule	13	2	5	7	0	3	3
	Mbale	10	2	0	7	0	3	5
	New Kawogo	6	0	2	2	0	5	5
	Others	17	0	7	5	0	5	0

The study shows that sweetpotato varieties susceptible to insect pests are cultivated by farmers across the six districts. This is due to the fact that high resistance to either *A. acerata* or *Cylas* spp. has not been found in the world sweetpotato germplasm collection (Mwanga et al. 2009), which could have been used to develop and release resistant varieties in Uganda. Evaluations for resistance against *C. puncticollis* of transgenic sweetpotato expressing Cry3Ca1, Cry7Aa1 and ET33-34 proteins from *Bacillus thuringiensis* (*Bt*) at the National Crop Resources Research Institute, Namulonge, Uganda are underway (Rukarwa et al. 2013).

## Conclusion and outlook

This study has provided insight into sweetpotato production in the main production districts of Uganda. Insect pests are a major production constraint in all the districts surveyed. The majority of farmers perceive insect pests to be a very serious problem. With the exception of Masindi and Wakiso districts where *A. acerata* is the number one constraint, sweetpotato weevils (*C. puncticollis* and *C. brunneus*) are ranked as the most important insect pests. However, many sweetpotato farmers take no measures to control *Cylas* spp. but invest in the use of chemical insecticides to control *A. acerata*, which has a high priority in Masindi and Wakiso districts. Promoting the use of cultural control methods such as mulching, re-hilling to cover soil cracks, use of clean planting material, crop rotation, taking time period and crops planted before into account, have potential to reduce damage by *Cylas* spp. Further, it would be important to invest in research to develop additional control measures. Biological control using the entomopathogen *Beauveria bassiana* has shown to reduce the *Cylas formicarius* Fabricius damage in Cuba alone or in combination with sexual pheromones (Lagnaoui et al. 2000) Farmers lack knowledge on *Cylas* spp. biology, damage and control. Hence, training about the biology and ecology of this important pest can help farmers to make informed crop management decisions. Baseline information gained from this study will assist the international agricultural research system (NARS), and non-governmental organizations (NGO’s) in designing IPM strategies that are based on the needs of smallholder farmers and their sweetpotato production systems. This information will also be useful in setting research priorities.
